# Biofabrication Strategies for Musculoskeletal Disorders: Evolution towards Clinical Applications

**DOI:** 10.3390/bioengineering8090123

**Published:** 2021-09-10

**Authors:** Saman Naghieh, Gabriella Lindberg, Maryam Tamaddon, Chaozong Liu

**Affiliations:** 1 Division of Biomedical Engineering, University of Saskatchewan, Saskatoon, SK S7N 5A9, Canada; 2 Christchurch Regenerative Medicine and Tissue Engineering (CReaTE) Group, Department of Orthopaedic Surgery, University of Otago Christchurch, Christchurch 8011, New Zealand; 3 Knight Campus for Accelerating Scientific Impact, University of Oregon, Eugene, OR 97403, USA; 4 Institute of Orthopaedic & Musculoskeletal Science, Royal National Orthopaedic Hospital, University College London, Stanmore HA7 4LP, UK

**Keywords:** additive manufacturing, 3D bioprinting, smart hydrogels, biomaterials, tissue engineering, musculoskeletal disorders

## Abstract

Biofabrication has emerged as an attractive strategy to personalise medical care and provide new treatments for common organ damage or diseases. While it has made impactful headway in e.g., skin grafting, drug testing and cancer research purposes, its application to treat musculoskeletal tissue disorders in a clinical setting remains scarce. Albeit with several in vitro breakthroughs over the past decade, standard musculoskeletal treatments are still limited to palliative care or surgical interventions with limited long-term effects and biological functionality. To better understand this lack of translation, it is important to study connections between basic science challenges and developments with translational hurdles and evolving frameworks for this fully disruptive technology that is biofabrication. This review paper thus looks closely at the processing stage of biofabrication, specifically at the bioinks suitable for musculoskeletal tissue fabrication and their trends of usage. This includes underlying composite bioink strategies to address the shortfalls of sole biomaterials. We also review recent advances made to overcome long-standing challenges in the field of biofabrication, namely bioprinting of low-viscosity bioinks, controlled delivery of growth factors, and the fabrication of spatially graded biological and structural scaffolds to help biofabricate more clinically relevant constructs. We further explore the clinical application of biofabricated musculoskeletal structures, regulatory pathways, and challenges for clinical translation, while identifying the opportunities that currently lie closest to clinical translation. In this article, we consider the next era of biofabrication and the overarching challenges that need to be addressed to reach clinical relevance.

## 1. Introduction

The musculoskeletal system, consisting of different types of bones, muscles, ligaments and tendons, is one of the key systems in the human body. According to the World Health Organisation, approximately 1.71 billion people suffer from musculoskeletal conditions which makes it a leading cause of pain and disability worldwide [1]. Musculoskeletal soft tissues are integral in ensuring joint movement and stabilisation. Injuries to these structures are prevalent, particularly in sports-active adults [2], and can be acute as a result of a traumatic event, or chronic as a result of overuse or cumulative trauma injuries [2,3]. Ruptures of the Achilles tendon and anterior cruciate ligament (ACL) are examples of acute injuries, which are among the most frequent and severe injuries sustained in a sports-active population [4,5]. Cartilage is another tissue that can be damaged as a result of a trauma, which, if not treated properly, can lead to degenerative diseases of the joints such as osteoarthritis [6].

Treatment modalities for musculoskeletal soft-tissue injuries are broad and range from “wait-and-see” approaches through to surgery [2]. Surgical treatments will depend on the type of tissue and the injury sustained; for example, in ACL reconstruction, the use of grafts (auto-, allo- and synthetic), specifically biological grafts, are considered the gold standard [4]. For cartilage defects, microfracture is used, where small holes are drilled into the subchondral bone to allow the transport of blood and bone marrow to the bone/cartilage interface, where the mesenchymal stem cells contribute to the formation and repair of the cartilage and bone [6]. Osteochondral auto- and allograft are other options for small- to mid-sized defects [6,7]. However, the current clinical methods of repair for musculoskeletal soft tissues are not without their drawbacks. For example, in cartilage repair using the microfracture technique, the regenerated tissue is fibrocartilage, which does not have the same durability as the native hyaline cartilage, leading to an ongoing articular surface irregularity and subsequent secondary arthritic changes [6,8]. A lack of tissue integration in the osteochondral graft technique is a major cause of failure in these operations [9]. In the autograft method of ACL reconstruction, tendons from other parts of the body, generally patella or hamstring, are used, which can lead to donor site morbidity and pain. Allografts have a risk of disease transmission and immune-mediated graft rejection [10,11]. Since they lack cellularity, they often require an extended period of revascularisation and incorporation (over a year) before a sports-active patient is allowed to return to play [12].

**Figure 1:**
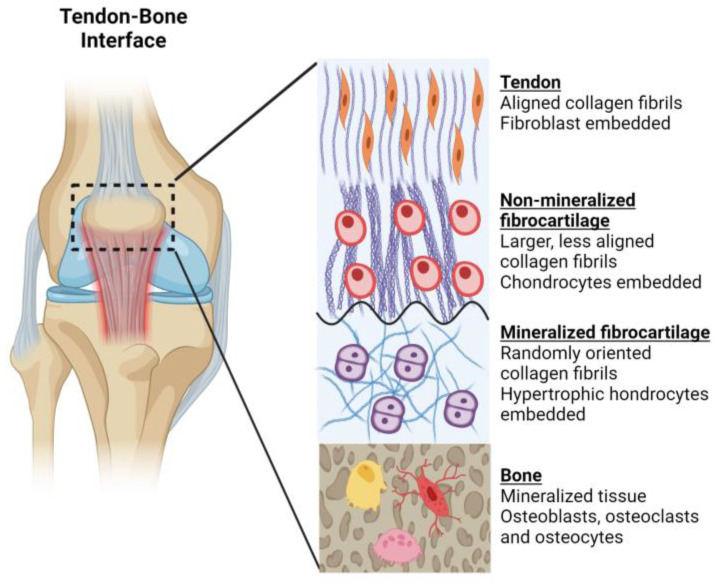
Tendon–bone interface showing the multicomponent and heterogeneous tissue structure. Adapted from Barajaa et al., 2020. Reused with permission from Springer Nature. Created with BioRender.com.

Considering the shortcomings associated with tissue grafts and other reconstructive options, tissue engineering approaches have been developed as an alternative treatment showing enormous potential for the regeneration of musculoskeletal soft tissues. However, their long-term success has been restricted, mainly due to the limitation in current fabrication methods that allows the production of a scaffold that mimics the cellular microenvironment and cell–cell interactions [13] observed in the native tissue. Musculoskeletal tissues are complex units, with compositional, mechanical, structural and cellular heterogeneity. For example, ligaments and tendons are connective tissues comprised of a dense band of aligned collagen (mainly type I) with embedded resident cells (fibroblasts/tenocytes) that connect bone to bone and muscle to bone, respectively [14,15]. The attachments between these interfacing tissues are unique and complex, with well-defined spatial changes in cell phenotype, matrix composition and mechanical properties (Figure 1) [14,16]. In the case of tendons/ligaments, the entheses (insertion into the bone) is composed of four distinct zones along the longitudinal direction: tendon/ligament (I), non-mineralised fibrocartilage (II), mineralised fibrocartilage (III), and bone (IV) [17,18]. While tenocytes/fibroblasts are embedded in the tendon/ligament matrix, the resident cells in the non-mineralised fibrocartilage are fibrochondrocytes, and hypertrophic chondrocytes are found in the mineralised fibrocartilage [19].

Considering the intricacies of the musculoskeletal tissues and the inherent heterogeneity in cellular and structural properties, it becomes clear that for a successful tissue engineering approach, the fabrication technique needs to recapitulate the compositional, mechanical, structural and cellular heterogeneity. Advances in additive manufacturing have opened new possibilities to fabricate structures with synergistic biological and mechanical properties that can mimic the natural tissue structures. Specifically, biofabrication or 3D bioprinting has been increasingly used as a revolutionary approach to healthcare that utilises additive manufacturing processes to produce a biological construct [20,21].

Biofabrication combines cells, biomaterials and biological factors—collectively known as bioinks—and delivers them in a precise pattern to recapitulate elements of heterogeneous tissues [22,23,24]. Although biofabrication has made a remarkable impact in areas of tissue engineering and drug delivery, its application and rapid progress into the clinic has been limited, firstly, by the lack of suitable biomaterials and bioinks [25,26]. In the field of orthopaedics, while a wide range of biomaterials have been applied as implants for treating common musculoskeletal defects, ranging from mechanically robust but relatively inert metals, ceramics and plastics [27], to bioactive but rather amorphous hydrogels [28], only a few are compatible with the process of bioprinting living cells [29,30,31,32,33,34]. In addition, although much research is being undertaken in the biofabrication field, few biofabricated constructs have yet been applied in widespread clinical settings due to a lack of regulatory clarity surrounding the frameworks in which these constructs lie.

This review paper thus looks closely at the processing stage of biofabrication with a focus on bioinks suitable for musculoskeletal tissue fabrication. We mainly explore synthetic and natural bioinks before discussing the underlying composite bioink strategies to address the shortfalls of sole biomaterials. We also review recent advances made to overcome long-standing challenges in the field of biofabrication, namely the bioprinting of low-viscosity bioinks, controlled delivery of growth factors, and the fabrication of spatially graded biological and structural scaffolds to help biofabricate more clinically relevant constructs. We further explore the regulatory and translational pathways for the clinical application of biofabricated musculoskeletal structures, highlighting both the challenges and the opportunities that currently lie closest to clinical translation. In this article, we consider the next era of biofabrication and the overarching challenges that need to be addressed to reach clinical relevance.

## 2. Biofabrication

**Figure 2:**
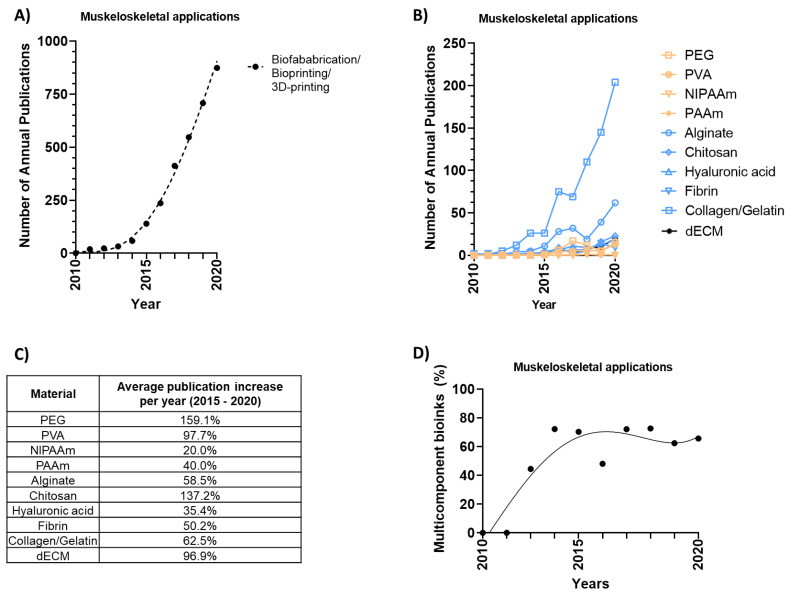
Analysis of publications within the field of musculoskeletal biofabrication over the past decade. An exponential increase in the total number of publications can be observed between the years 2010 and 2020 (**A**). There is a preference for using naturally derived polymers as bioink components over synthetic materials (**B**). While the use of both collagen/gelatin and alginate-based bioinks has generated the highest interest overall, the use of both PEGs and chitosan has increased the most in the past 5 years (**C**). The field has steadily moved from single component bioink formulations to more complex multicomponent structures (**D**).

Additive manufacturing can be categorised under three groups of laser/light-based, extrusion-based, and jetting/powder-based technologies. Specifically, additive manufacturing methods have been developed recently for biofabrication applications under these three groups. Additive manufactured biofabrication methods are lithography-based (light/laser-based group), extrusion-based, and inkjet/multi-jet bioprinting (Figure 2). In biofabrication, complex, sophisticated and biomimetic tissues can be constructed with high precision. Another advantage of this method is the ability for automation and control over geometrical intricacies. This includes the possibility of incorporation and precise spatiotemporal placement of cells, proteins, drugs and biologics [35].

Among biofabrication methods (Figure 2), stereolithography (SLA), also called digital light processing, is known as one of the oldest additive manufacturing techniques. Due to its relatively high precision, it is commonly used to create anatomical models for preoperative planning [36]. In this method, photosensitive resin is exposed to a high-intensity light source in order to solidify and fabricate a 3D structure layer-by-layer. One of the advantages of this technique is that the fabricated structure has a clear to opaque white appearance after fabrication due to the nature of utilised photopolymer materials; this can help to visualise internal anatomies clearly. In addition, photosensitive materials are limited, and as a result, it limits the use of this method [29,30]. Extrusion-based bioprinting is operationally more adoptable with respect to printing multiple biomaterials and cell types and it has opened new ways to fabricate complex tissues and organs [37]. Specifically, this technique has been used widely to create hybrid and composite structures in order to have both hydrogels and synthetic polymers in one structure; hydrogel creates a cell-friendly environment used for drug-delivery purposes and growth factor/cell incorporation, while a synthetic polymer is used to enhance the mechanical properties due to the poor mechanical characteristics of hydrogels.

In bioprinting, cell-laden bioinks are fabricated into a functional tissue from 3D digital models [35]. In general, there are three major stages in biofabrication: preprocessing, processing and postprocessing [35], each of which can be further divided into smaller steps. In preprocessing, a digital model is generated from imaging (X-ray, computed tomography (CT), magnetic resonance imaging MRI) and the design approach (biomimicry, self-assembly, mini-tissues) is selected. In the processing stage, the bioink material (natural polymers, synthetic polymers, ECM), cells (differentiated cells, pluripotent stem cells, multipotent stem cells) and bioprinting technique (lithography-based, extrusion-based, and inkjet/multi-jet bioprinting) are selected. Finally, in the postprocessing steps, according to the application (maturation, implantation, in vitro testing), the tissue is utilised [38].

## 3. Designing Musculoskeletal Bioinks

Significant breakthroughs in material synthesis and crosslinking chemistry over the past decade have enabled the biofabrication of precursory solutions consisting of both living components and crosslinkable polymers, so-called bioinks [30,33,39,40,41,42,43,44,45,46,47,48,49,50,51,52,53,54,55,56]. These polymers can hold large amounts of water once crosslinked into a hydrogel network [57,58], which is key to protecting both the cells and biological compounds during and after the biofabrication process. These bioinks can be fabricated from both biological and synthetic polymer materials, having more or less inherent biocomplexity, which comes with different sets of both benefits and limitations. Simplified, synthetic polymers offer more precise control over the system but lack the necessary and appropriate biological cues to help guide cell development, and vice versa. However, the shortcomings of both classes of materials are nowadays, to a certain degree, possible to overcome by appropriate chemical and biological modifications as well as hybrid technologies [29,59]. In this section, we will look more closely at the choice of bioinks used for musculoskeletal soft tissue fabrication and the clinical relevance of the different classes of materials.

A total of 4105 publications (out of which 1222 are review articles) containing the keyword “3D-printing”, or “3D-bioprinting”, or “biofabrication”, and either “bone”, “cartilage”, “osteochondral”, “tendon”, “muscle”, “osteogenesis”, “chondrogenesis”, or “vasculogenesis”, and not “cardiac”, were retrieved from the Scopus database from 2010 to 2020, disclosing an exponential growth with an average publication increase of 53.3% per year (Figure 2A). The retrieved data reveals that the use of naturally derived materials far outnumbers synthetic based bioink systems throughout the analysed time-period. As further detailed in Figure 2B, the number of original research articles exemplified by some of the most commonly applied material types confirms that both collagen/gelatin and alginate-based bioinks have remained a popular choice over the past decade (876 publications combined). While an increase in publications has been seen across all material types, it should be noted that the use of both polyethylene glycol (PEG, 159.1%) and chitosan (137.2%) have increased the most over the past 5 years (Figure 2C). This may reflect that many crosslinking systems can benefit from the added control that synthetic PEG-based linkers provide, while the addition of naturally derived chitosan offers a biofunctional strategy to increase the viscosity of various bioink formulations. While early biofabrication studies focused on the development of single-component bioink formulations, the field quickly recognised the power of hybrid bioink formulations and shifted towards multicomponent bioinks. Between the years 2010 and 2013, 82% of the published papers were based on single-component bioink formulations, while the majority of papers (67%) published between 2014 and 2020 utilised multicomponent bioinks (Figure 2D). More detailed information around the use of these commonly applied bioink materials are exemplified in the sections below.

### 3.1. Synthetic Materials

The application of synthetic polymers as shear-thinning bioinks has proven advantageous as it comes with high printability, structural fidelity, batch reproducibility, and industrial scalability [31,32,33,60,61,62]. Synthetic bioinks have thus been used since the beginning of the biofabrication era. Key examples includes the use of Polyethylene glycols (PEGs) [63,64,65,66], Poly(vinyl alcohol) (PVA) [46,67,68,69], N-isopropylacrylamide (NIPAAm) [70,71], and polyacrylamide (PAAm) [72,73,74]. In this section, recent developments and clinical advantages of these materials are highlighted.

**Figure 3:**
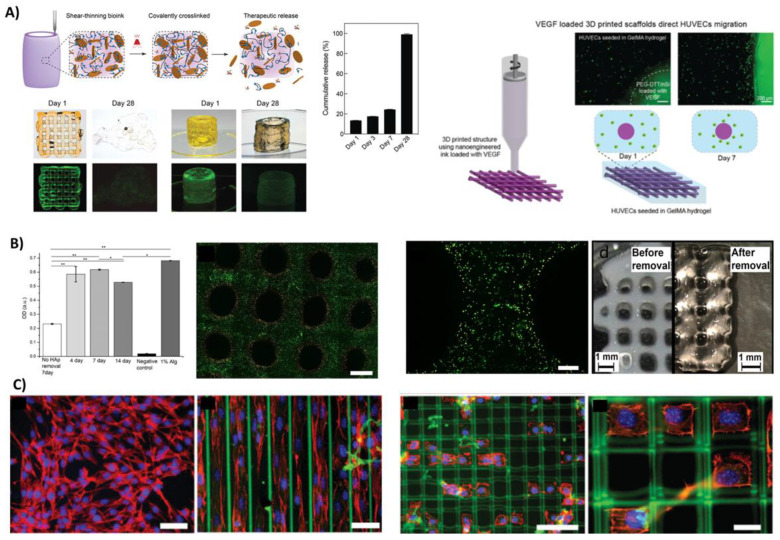
Key examples of synthetic materials used as bioinks and biomaterial-inks. By tuning the function of degradable PEG-based bioinks and the biofabrication of high surface area structures using extrusion-based printing technologies and UV crosslinking, Peak et al. prolonged the delivery of VEGF for up to 28 days to direct the migration of HUVECs encapsulated in surrounding GelMA hydrogels after 7 days of culture (**A**). Change the format as follows: Reprinted with permission from ref. [63]. Copyright 2019 John Wiley and Sons. PNIPAAM-based bioinks printed with extrusion-based technologies show better chondrocyte viability after washing away residual pNIPAAM, with increasing cell death observed around the pores, * *p* < 0.05 and ** *p* < 0.001 (**B**). Change the format as follows: Reprinted with permission from ref [70]. Copyright 2015 Acta Biomaterialia. Other examples include acrylamide and glycerol-based biomaterial inks extruded with directional control to enable alignment of 3T3 fibroblasts using a microperiodic hydrogel scaffold (green) as compared to the flat glass control (left, **C**). Reprinted with permission from ref [73]. Copyright 2009 John Wiley and Sons.

By optimising crosslinking concentrations, Peak et al. was able to tune the function of degradable PEG-based bioinks to prolong the delivery of growth factors for up to 28 days and thus promoted the migration of endothelial umbilical vein cells for long-term cultures, as seen in Figure 3A [63]. This directed chemotaxis strategy holds great translational value as the retained 4-week bioactivity allows for reduced growth factor concentrations to be used. Furthermore, it can be used as a cell-free, and thus an off-the-shelf, product with tailorable release profiles. In addition to delivering therapeutics, PEG can be designed to have a broad range of molecular weight distributions and functional arms for chemical crosslinking, explaining its widespread use across various platforms [75,76,77]. While having a similar chemical structure, additional side-groups provided through Poly(glycidol)s (PG) based bioinks enable even further tailorability, albeit still requiring the addition of naturally derived biomaterials to induce cellular functionality [62].

Other synthetic materials like PVA can be used as both a core component of a bioink formulation [67] as well as a sacrificial template to support the initial fabrication process [78]. By depositing sacrificial PVA fibres, multilayered biomaterial-ink constructs with clinically relevant dimensions can be fabricated [78]. These scaffolds may be applied as cell-free products, with a shorter path to the clinic. Using clever chemistry modification strategies, PVA hydrogels can furthermore be designed to be hydrolytically degradable to help control the delivery of bioactive factors to surrounding cells [68]. Musculoskeletal factors such as VEGF, bFGF and BMP can be delivered with tailorable long-term release profiles, reaching over three months of sustained release. Enabling prolonged growth factor release holds great power as it can sustain bioactive factors throughout the musculoskeletal healing processes and not just the initiation. The optimal therapeutic window is furthermore known to be very different for e.g., VEGF and BMP [79], which may nowadays be possible to recreate with these recent developments of tailorable and sustained bioink delivery platforms.

NIPAAm is another example of a synthetic polymer used as a bioink, but it has reverse thermoresponsive properties. It remains liquid below 32 °C and solidifies at higher temperatures [80]. This enables easy mixing and loading at room temperature, while it can be 3D printed onto a heated surface where it solidifies. Although its “on-off” gelling properties can be useful, its network formation is however highly affected by salt concentrations [81] it furthermore exhibits complex synthesis routes [71,82], which limit its universal use. It is also critical to remove the NIPAAm components post printing to ensure good cell viability [70], making it an inappropriate material to work with for bioprinting applications. As seen in Figure 3B, even structures that have washed away NIPAAm post printing demonstrate reduced viability around the pores where the sacrificial NIPAAm was deposited during printing. Additional examples of synthetic bioinks include PAAm blends that can be used to guide cellular growth with high accuracy and directional input [72,73,74]. This is an interesting concept as it allows users to align cellular growth and direct the interaction between neighboring cells, as seen in Figure 3C. This ability may be of particular interest for musculoskeletal applications as the cellular and matrix orientation is very distinct across different zones within musculoskeletal soft tissues [83,84,85,86]. Furthermore, the mechanical properties of PAAm can be tailored to facilitate soft–hard interfaces required within musculoskeletal regeneration [74]. This capability is highly important for musculoskeletal systems, which are comprised of three distinct tissue properties and the interfaces between them: (1) hard mineralised tissues, (2) soft muscular tissues, and (3) viscoelastic connective tissues [87].

While several impactful advances have been made in the past decade, the evident common denominator that still confines the clinical applicability of synthetic bioinks is their limited interactive capacity with cells and the requirement of additional biologicals to successfully direct the musculoskeletal development and prevent cell death. Finding the right formulations of bioactives to deliver with these synthetic bioinks is a tedious task, which ends up introducing the very biological variance that synthetic bioinks are designed to reduce. Nonetheless, synthetic formulations play a key role in personalised medicine as they are easily modified and provide low to no immunogenicity with relatively high controllability [88]. It should, however, be noted that antibodies against synthetic biomaterials have been reported [89] and should thus be fully considered and evaluated prior to any clinical translation, especially for chemically modified synthetic polymers that undergo in situ crosslinking during the bioprinting process.

### 3.2. Natural Materials

The application of naturally derived polymers as bioinks is attractive as it includes motifs that are recognised by cells and can be used to guide the tissue regeneration process. However, natural hydrogels that can be fabricated on a kg-scale at a low price, narrows down the library of possible candidates. Commonly used candidates are thus centred on alginate, chitosan, hyaluronic acid (HA), fibrin, and collagen/gelatin [39,42,49,90,91,92,93,94,95,96,97,98,99,100,101,102,103,104,105,106,107,108,109]. Additional work has been performed with decellularised materials as it offers additional biological complexity and stimulation. With increased complexity, however, comes larger batch-to-batch variation, a shorter shelf-life and unpredictable outcomes. In this section, the applicability of these materials as bioinks and their clinical advantages are highlighted.

Alginate is derived from brown seaweed and is an anionic polysaccharide that contains carboxylic acid groups, which allows ionic crosslinking following exposure to calcium chloride [90,107,110]. The molecular weight distributions of alginate and material concentrations can be easily modulated to tune the viscosity, and subsequently, printability, as well as the mechanical properties and degradability to direct cellular responses [90]. Alginate bioinks can be used as both permanent structures [91,92,93,94] or as sacrificial templates [95,96]. Alginate is furthermore often used with the method of freeform reversible embedding of suspended hydrogels (FRESH) [111,112,113]. Hinton et al. has demonstrated that alginate can be deposited within a thermoreversible support bath, such as gelatin, to fabricate femurs and vascularised structures of clinically relevant sizes [113]. This strategy is interesting as it can generate hollow structures with ease because it is not limited by a layer-by-layer planar fabrication process. Furthermore, it is easy to print liquid bioink formulations at room temperature [112]. While this strategy further allows the fabrication of large volume structures, the resolution of the printed structures will require further optimisation as it is often restricted to >100 µm [112]. When comparing alginate to other naturally derived bioinks, Demirtaş et al. found chitosan to be superior to alginate when encapsulating MC3T3-E1 pre-osteoblasts [91]. Chitosan has recently been applied as a bioink for 4D printing, allowing a change in structural properties over time [102]. Due to the dynamic and reversible movement of water within the chitosan-based bioink as a function of temperature-induced pores, Seo et al. were able to tune the shape of the implants [102]. This reversible shape-morphing is highly interesting from a gradient and stimuli-responsive design aspect. However, the field is still in its infancy, and most current developments still focus on designing bioinks that exhibit an adequate response to stimuli [114].

**Figure 4:**
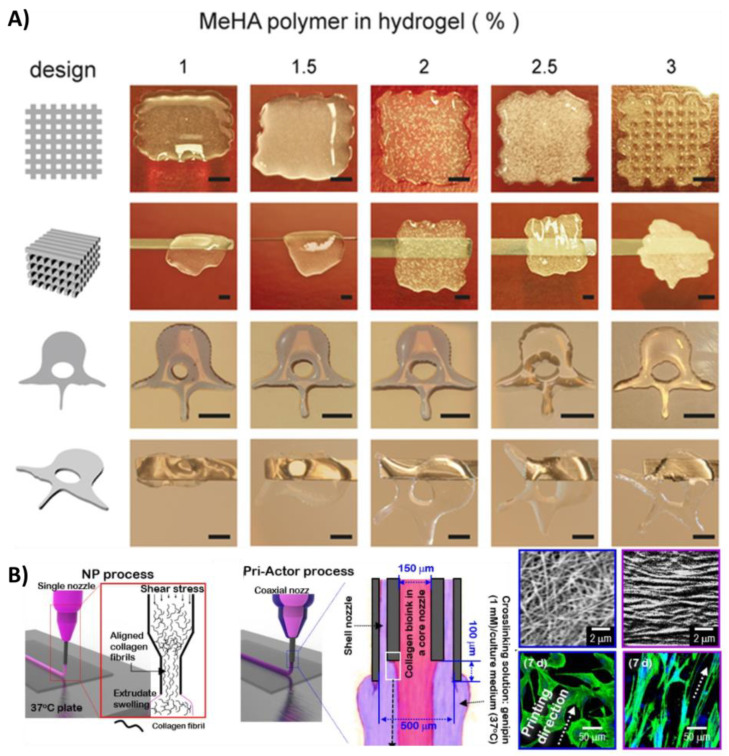
Examples of naturally derived bioink applications. Using hyaluronic acid and extrusion-based printing technologies, designs of both porous cubes and non-porous L3 vertebras can be achieved with good shape fidelity and tissue regeneration properties (**A**). Reprinted with permission from ref. [115]. Printing collagen fibres with a Pri-Actor system enables the hydrostatic forces to align the fibrils and subsequently the orientation of F-actin of encapsulated hASCs (**B**). Reprinted with permission from ref. [140].

Demirtaş et al. furthermore reported that the addition of HA was a key component that significantly improved the cellular performance across both alginate and chitosan-based bioinks [91]. As exemplified in Figure 4A, HA has been widely applied for musculoskeletal applications [115] as it furthermore displays a low toxicity and inflammatory response, which are key aspects for clinical translation [116]. HA has the proven ability to direct vascularisation, osteogenesis, chondrogenesis and cell migration, while providing structural integrity post printing [44,107,116,117,118,119,120]. HA is, however, not thermoresponsive, which can either be an advantage or disadvantage depending on the bioprinting technology used. Although HA has shown great potential over the years, amounting to many commercial products, it is still costly to fabricate [121] and is thus often applied as a supplement with other bioink materials. Only 5% of HA-containing bioink publications were pure HA formulations (single component) in the year 2020.

While fibrin is an exciting base material that has proven very useful in clinical trials to repair musculoskeletal tissue—demonstrating both improved bone mineral density and osteocalcin levels [122], when combined with biofabrication technologies it is today mostly used for cardiac and neural regeneration [38]. This gap might be explained by the lack of structural stability provided when applying fibrin-based bioinks, which is a prerequisite for biofabricating load bearing tissues. Due to its low viscosity, it is mostly confined to inkjet printing which further limits its widespread use, as the shape fidelity and mechanical properties of such biofabricated structures are low [105]. To overcome this limitation, fibrin is more commonly used as a bioactive component added to 3D-printed muskuloskeletal implants for load bearing applications and has shown promising results to decrease the need of autologous bone transplantation [123]. Fibrin hydrogels have also been designed to have high elasticity and large network gaps to bind and release growth factors for up to 21 days and subsequently promote bone tissue repair. So, the clinical potential of fibrin is already well established, and when it comes to musculoskeletal applications, is currently preferred as a supplement post the biofabrication process rather than as a bioink.

As seen in Figure 2, collagen and gelatin materials have long since been a preferred choice for many of those designing bioinks, and have been recognised to play a critical role in the acceleration of the clinical translation of biofabricated musculoskeletal implants [124]. Collagen, and its irreversibly hydrolyzed form gelatin, is a water-soluble protein often derived from porcine or bovine sources, but has recently gained interest by being sourced from other animals including fish [125]. Several examples have highlighted the potential of both collagen and gelatin-based bioinks [39,45,46,49,109,124,126,127,128], most commonly combined with extrusion-based technologies due to their thermoresponsive properties [124,129]. Although both collagen- and gelatin-based medical devices have long since been used within clinics to repair damaged tissues [124,130,131,132,133,134,135,136,137], it is still far from the standard musculoskeletal repair practice, as it is not consistently successful. Problems with limited tissue regeneration, the formation of mechanically-inferior fibrocartilage and a poor integration with the native tissue persist, resulting in implant failure for most patients [138,139]. On a global scale, treatment failure of knee-specific musculoskeletal injuries is often correlated with larger defect sizes, increasing from a 4.3% failure rate in less complex cases to 87.5% in advanced end-stage osteoarthritis cases [139]. To overcome these limitations, it is thus important to design large-scale collagen/gelatin implants with more biomimetic architectures. To this end, Kim et al. combined a collagen- based bioprinting process with an in situ bioreactor system to regenerate muscle tissue [140]. By controlling the mechanical stresses generated from the bioink flow, the coaxial in situ bioprinting technology demonstrated alignment of the collagen fibrils as compared to randomised orientations following a traditional extrusion bioprinting setup, as seen in Figure 4B [140]. This biochemical and biophysical stimulation was able to induce osteogenic differentiation without any additional growth medium. It was furthermore reported that myofibres were formed densely, while reduced fibrosis was observed within these novel structures following a five week in vivo implantation [140].

While this exemplifies an elegant strategy to improve structural biomimicry in collagen/gelatin bioinks, researchers still struggle to meet regulatory constraints as these bioinks are still known to contain highly variable levels of endotoxins [141].

### 3.3. Composite Materials

To overcome some of the above-listed limitations of individual materials and improve the biological relevance and translational power, composite bioinks have long since attracted attention. While several dual networks have been developed [61,98,104,107,110], new formulations are starting to evolve, consisting of three or even four components to improve bioink properties and, subsequently, the functionality of musculoskeletal bioinks. Specifically, this section highlights some of the main composite strategies developed to better balance the controlled bioactivity and mechanical properties of biofabricated structures.

**Figure 5:**
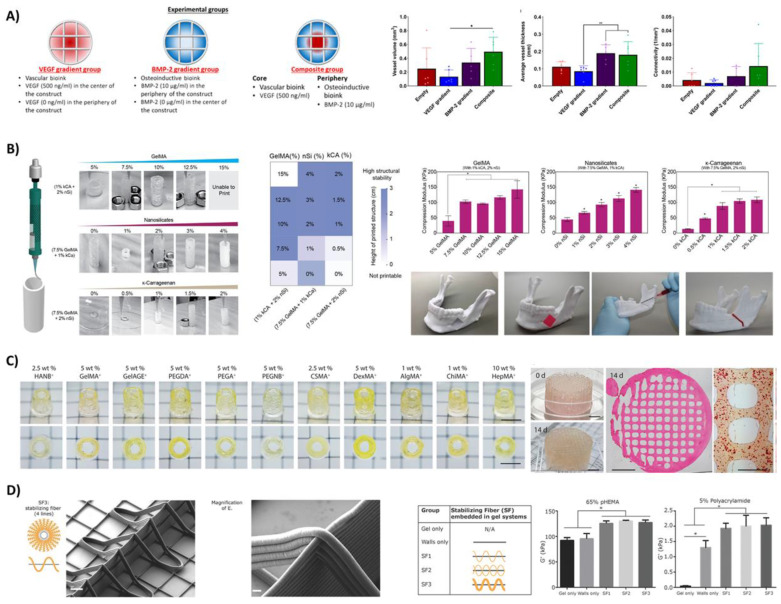
Examples of hybrid bioink strategies. Using dual extrusion technology to generate core–shell structures of nanoparticle functionalised alginate bioinks promotes vessel volume, vessel thickness as well as connectivity of encapsulated HUVECs, * *p* < 0.05 and ** *p* < 0.001 (**A**). Reprinted with permission from ref. [143]. Extrusion of hybrid bioinks made from combinations of GelMA, kappa-carrageenan, and nanosilicates enables a wide biofabrication window to generate clinically relevant and resilient constructs, * *p* < 0.05 (**B**). Reprinted with permission from ref. [147]. Three dimensional printed tubular constructs made of a variety of hyaluronic acid, gelatin, PEG, chondroitin sulphate, dextran, alginate, and heparin mixtures allows the maintenance of Saos-2 cells with osteogenic mineralisation (**C**). Reprinted with permission from ref. [98]. Other examples include adding stabilising polyacrylamide fibres in poly(2-hydroxyethyl methacrylate) mixtures, printing various orientations to tailor the mechanical properties, and increasing the complex shear modulus of implants, * *p* < 0.05 (**D**). Reprinted with permission from ref. [156].

Composite examples with more than two components often include heparin conjugated bioinks as these can sequester growth factors tethered into the bioink network [142]. Other alternatives include laponite-infused bioinks, which can also sequester several growth factors within multicomponent 3D-hydrogel structures [143]. Freeman et al. were able to use this strategy to control both the temporal and spatial release of VEGF and BMP, demonstrating an increase in vessel infiltration as compared to homogeneous distribution within bulk structures with the same growth factor concentrations, as seen in Figure 5A [143]. These studies that utilise the synergistic effect of dual growth factor delivery are key to advancing the clinical relevance of bioinks. This tight control of growth factor delivery within optimal therapeutic windows is a prerequisite to successfully fabricating implants with low therapeutic dosages, reducing the risk of adverse effects often associated with burst releases and the subsequent need for supraphysiological doses [144], which is further discussed in Section 3.4.2.

Other multicomponent bioink formulations focus on the incorporation of nanoparticles [60,145,146]. Chimene and colleagues, for example, were able to tune both the mechanical and biological activity of multicomponent bioinks consisting of gelMA to induce endochondral differentiation even in the absence of osteoinductive agents, as seen in Figure 5B [147]. The ability to induce cellular differentiation without the supply of optimised media components in vitro is an important stepping stone to translating developed therapies to a clinical setting, where the supply of nutrients is sparse, and surrounding cells are often residing in a diseased and subsequently inflamed environment. By incorporating hydroxyapatite nanoparticles into a gelatin/PVA bioink mixture and further reinforcing the structures with PCL, Kim et al. were similarly able to promote calcium deposition and ALP activity as compared to the two-component control structures using gelatin/PVA mixtures alone [69]. This highlights the need to move to more complex formulations containing more than two-component mixtures of materials to guide the musculoskeletal tissue regeneration process better.

An interesting multicomponent example is furthermore provided by Leucht et al., demonstrating how the base material can remain the same, and the bioink flexibility is instead provided through alternative chemical modification strategies, using blends of unmodified gelatin, methacryloyl-modified gelatin and acetylated gelatin [43]. By providing gelatin bioink with various modifications, the team was able to support the interplay between vasculogenesis and osteogenesis. The sacrificial nature of including nonmodified gelatin allowed the authors to reap the rewards of both improved printability during the fabrication process and reduced crosslinking density and elevated swelling, which are key factors for promoting the formation of vascularised tissues. Similarly, Ouyang et al. demonstrated that unmodified gelatin can be used to obtain a wide range of printable bioinks [98]. Using a collection of chemical modification protocols and a library of base materials, including gelatin, hyaluronic acid, chondroitin sulfate, dextran, alginate, chitosan, heparin, and poly(ethylene glycol), the authors were able to fabricate complex structures from soft bioinks that were able to support the musculoskeletal mineralisation process, as the mechanical properties remained within the optimised window following the addition of unmodified gelatin, as seen in Figure 5C [98].

Advances in the past decade further highlight the potential of hybrid approaches to combine soft and biomimetic hydrogels with synthetic and mechanically robust thermoplastic materials [29,104,148,149,150,151,152,153,154]. Pioneering this interpenetrating network strategy, Visser et al. and Kang et al. showcased that the mechanical properties of hydrogels can increase 54-fold, approaching near-native cartilage properties [154], and can be fabricated into perusable structures with good mechanical integrity following PCL reinforcement [155]. By advancing the resolution to fabricate well-defined biphasic structures, de Reuitjer et al. were able to address mechanical properties beyond just the compression strength. By introducing cross-printed fibres, so-called “out-of-plane” printing, additional control over shear and tensile stresses are introduced to withstand the forces from everyday life better, as seen in Figure 5D [156]. These emerging composite strategies can fulfill unmet clinical needs in musculoskeletal tissue engineering. They overcome many of the traditional trade-offs between mechanical support and biological function without compromise.

### 3.4. Functional Properties and Clinical Challenges of Musculoskeletal Bioinks

While in its infancy, the field of biofabrication was mainly focused on structural shape fidelity and cell viability [60]. Following the mapping of detailed processing and rheological requirements [60,157] and the subsequent development of a wide library of polymers compatible with biofabrication [29,30,32,158], the next era of biofabrication is now focused on structure–function relationships [31]. This includes formulating bioinks with controllable physiochemical and biological cues. The microenvironmental properties of the bioinks surrounding the encapsulated cells are major determinants of cellular development in 3D. In this section, we specifically step through the recent developments made in bioprinting of low-viscosity/soft matrices, the spatial control over growth factor delivery, and the bioprinting of anisotropic 3D cellular niches through high-resolution gradient structures for improved cell responses and more accurate physiological models of musculoskeletal tissues.

#### 3.4.1. Low-Viscosity Bioinks

It has long since been desirable to biofabricate structures using bioinks with a low polymer concentration as this can provide a suitable environment for cellular growth. Soft matrices can permit cell migration, vascularisation and diffusion of nutrients and regulatory molecules inside the scaffold. However, fabrication of a 3D-printed porous structure made of low-viscosity bioinks has traditionally been challenging as it was long centred on extrusion-based biofabrication [159]. As using higher polymer concentrations often leads to increased stiffness and subsequently reduced tissue outgrowth [160], alternative approaches using sacrificial templates and 3D-printed assisted molding technologies became a popular alternative for extrusion-based 3D bioprinting of nonviscous bioinks. Such a strategy is known as indirect bioprinting, a fabrication method used to create a sacrificial framework while printing the final structure of a scaffold [161]. This indirect technique opens the opportunity to create hybrid or composite structures with the assistance of different biomaterials. Furthermore, indirect bioprinting can realise scaffolds with advanced architecture as it allows for control over both the external and internal structure. The most popular strategy is to use Pluronic™ F-127 as a sacrificial ink, a reverse thermoresponsive polymer which liquefies at low temperatures and solidifies at higher temperatures with fast viscosity recovery after shearing [162,163,164]. Using this strategy, Kolesky and colleagues casted GelMA onto 3D-printed Pluronic F127 structures, leaving a perfusable channel for HUVEC endothelialisation after dissolution and removal of Pluronic F127 through submersion of the whole construct in a cool liquid [127]. Recent examples of using sacrificial inks to fabricate low-viscosity GelMA (≤5 wt.%) include multimaterial formulations, where the addition of alginate to the pluronic F127 reduced the osmotic interaction between inks and further improved the stability of the sacrificial ink. As such, it was possible to fabricate interconnected structures of both fast crosslinking gelatin methacrylate and slow crosslinking collagen type I [165]. It should however be noted that concerns have been raised around cellular toxicity following exposure to Pluronic F-127 [10]. Looking at alternative materials as sacrificial inks, Norotte et al. were able to fabricate hollow channels of multicellular pig smooth muscle cells using agarose fibres as a sacrificial ink [166].

Still, the resolution of the final structures is limited to the fibre thickness of the extruded pluronic ink (≥300 µm). Other strategies have thus been developed to overcome these extrusion-based resolution restrictions, such as in situ crosslinking of the bioinks. By crosslinking the bioink during extrusion, the low-viscosity bioink filaments were able to be deposited with good shape fidelity and viability [41,103]. This technique however, requires detailed optimisation of the timing of the light irradiation and its intensity. Other recent developments include the use of support baths for printing low-viscosity bioinks using extrusion technologies. The technique, known as (FRESH) enables printing of soft matrices that would otherwise collapse during traditional extrusion printing. Hinton et al. elegantly demonstrated biofabrication of alginate, collagen, and fibrin with an elastic modulus of <500 kPa and a resolution of approximately 200 μm using this strategy [113].

Biofabrication of low-viscosity bioinks can also be achieved with other technology platforms, such as inkjet and laser-based technologies SLA, DLP and volumetric printing. However, most of these technologies require the precursory solution to be completely liquid, which might also be hard to achieve within biologically compatible temperatures (≤37 °C) when using polymers with high molecular weights. By patterning alginate and ECM-based bioinks using inkjet technology, Negro et al. demonstrated that it is also possible to align several low-viscosity bioinks in a droplet jetting fashion to generate larger multicomponent hydrogel structures [167]. By mixing liquid formulations of PVA-MA and Gel-MA in a DLP printer, Lim and colleagues were able to fabricate structures with a 25–50 µm resolution without sedimentation of the encapsulated cells to promote chondrogenic and osteogenic differentiation [46]. Bernal et al. were able to speed up this light-initiated printing process of low-viscosity bioinks, generating free-floating structures using volumetric printing. This rapid fabrication speed holds an important clinical benefit, as it allows for the biofabrication of large constructs with an arbitrary shape within seconds, with the proven ability to support the deposition of matrix components present in the native meniscus tissue [45]. These advances highlight the importance of both advancing material formulations as well as technological toolkits to resolve some of the main challenges in the field of biofabrication.

#### 3.4.2. Controlled Delivery of Growth Factors and Cells

A key aspect to the successful regeneration of musculoskeletal tissues is the spatial–temporal presentation of bioactive components, specifically growth factors (GFs). While many recent advancements have demonstrated the capability to 3D-pattern GFs within biofabricated structures [101,168,169], several challenges still remain around controlled delivery and subsequently clinical translation. Fabricating growth-factor loaded constructs still comes with a traditional trade-off between maintained bioactivity and sustained release profiles. The most straightforward method to immobilise GFs in hydrogels is by physical entrapping, mixing the factors with the bioink. Although examples have been able to show maintained bioactivity using this strategy, for example, highlighting that both dual and triple delivery of GFs is possible, this physical entrapment approach is inefficient and unpredictable [170,171,172,173,174,175]. A burst release of GF is often seen, losing most of the factors after just hours or a few days [175]. So, although the chemical functionalisation of growth factors is well known to reduce the inherent bioactivity [176,177,178,179], often due to steric hindrance or structural modulation, it has been suggested that covalently bound growth factors can achieve more significant osteogenic differentiation as compared to physically entrapped bioactives due to improved retention over longer periods [59,170,173,174,179,180,181,182].

However, recent developments within biofabrication are now offering strategies to overcome this traditional balancing act between maintained bioactivity and sustained delivery. For instance, using 3D extrusion of core–shell fibres to physically immobilise GFs within bioinks, Akkineni and colleagues optimised the delivery of BSA, VEGF as well as BMP-2 from a 3-day burst release to a linear release profile over seven days [101]. Freeman et al. further demonstrated detailed delivery control of VEGF and BMP-2 to accelerate large bone defect healing [143]. The concentration needed for a good therapeutic response was significantly lowered (1–100-fold) in this specific study, as compared to other fabrication strategies [162,183,184,185], as the biofabrication technology enabled the codelivery of multiple GFs with good temporal control. Seeing good therapeutic effects with reduced GF concentrations is of high importance as GF-loaded hydrogels commonly fail clinical trials due to supraphysiological doses triggering several harmful side effects [145]. Additional examples include gradient delivery of BMP-2/TGF-β1 or BMP-2/VEGF combinations [168,169]. In the latter study [169], the synergistic osteogenic and vasculogenic effects following a slow BMP-2 and quick VEGF release allowed for 5 µg/mL and 2 µg/mL doses, respectively, which is well below the traditional 150–600 μg/mL range often required for human osteogenesis. Such strategies could serve to release the appropriate GFs with both accurate doses and kinetics and a key to advancing the clinical relevance of GF loaded hydrogels.

It should similarly be noted that designing bioinks that require low cell seeding densities is of equal importance, as clinically sized structures will otherwise require large amounts of healthy cells, which it may not be possible to obtain from an autologous harvest. To this end, Henrionnet et al. bioprinted a 10 × 10 × 4 mm construct with a seeding density of only 1 × 10^6^ cells/mL. Interestingly, the lower seeding density of 1 million cells was shown to induce Collagen II gene expression as compared to a higher concentration of 2 × 10^6^ cells/mL within the bioprinted alginate, gelatin, and fibrinogen matrix [186]. While very high cell concentrations may attribute limited oxygen and nutrient diffusion, and subsequently cell death, this appears not to be the case in this study as 2 × 10^6^ cells/mL is still a relatively low concentration. Instead, it may be considered that the native cell density in soft musculoskeletal tissues only represents 2% of the hyaline cartilage volume [187,188,189] and that a low concentration may be more biomimetic. In addition, by overcoming traditional limitations of cell harvesting and culture, bioinks that enable reduced cell numbers may further hold potential to the reduce risk for tumorigenesis and microembolism [190].

Biofabrication technologies further offer a unique platform to also control the spatial location of cells. A recent example of this includes Ker et al. utilising patterns of BMP-2 and FGF-2 for tendon–bone interface engineering [191]. Through inkjet technologies, the growth factors could be distributed onto oriented polystyrene fibres, which directed the differentiation of cells based on their location, enabling the formation of osteoblasts and tenocytes within different regions of the structure [191]. A similar example, using GelMA-based bioinks containing TGF-β1 and BMP-2 deposited as nanolitre droplets using inkjet printing, allowed for growth factor gradients to guide both osteogenic and chondrogenic differentiation within an implant [168]. It is thus evident that controlled delivery of both growth factors and cells are needed to help tune and control the functionality of clinically relevant constructs.

#### 3.4.3. Hierarchical Structures

**Figure 6:**
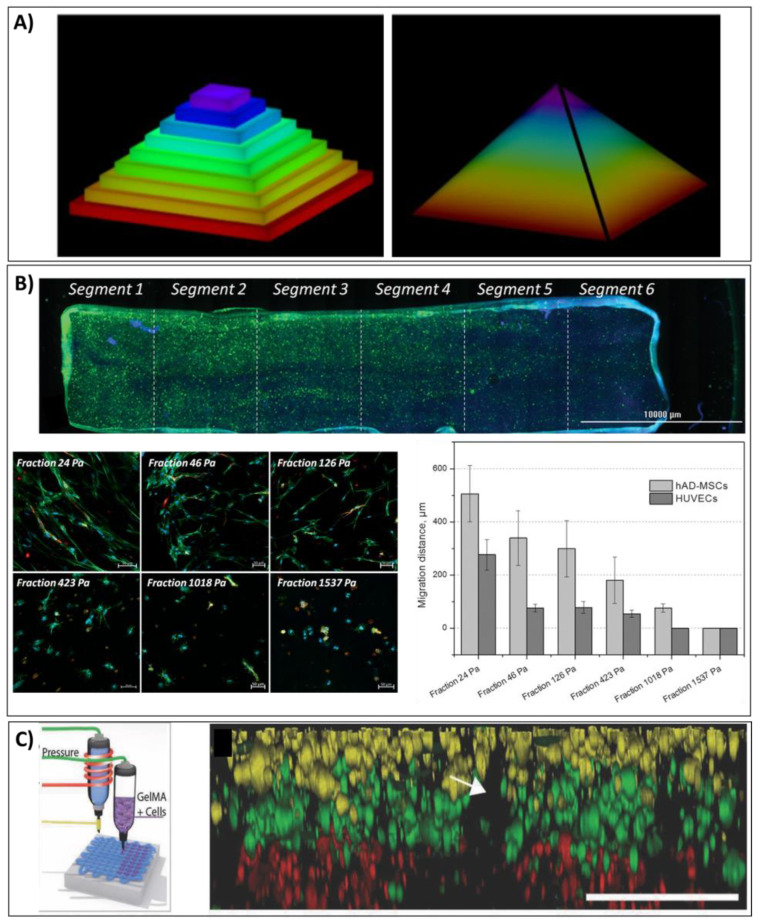
The new era of biofabrication is moving from low-resolution layer-by-layer stacking of objects with a limited volume and into high-resolution printing by decreasing the layer height to generate a smoother surface (**A**). Reprinted with permission from ref. [195]. Bioprinting of GelMA with a controlled stiffness gradient allows encapsulated HUVECs (blue tracker) and adipose derived MSCs (green tracker) to be encapsulated with spatial gradients (**B**). After 7 days of culture, immunohistology of f-actin (green), CD31 (red), and DAPI (blue) demonstrated that cells had very distinct morphologies depending on the stiffness of the local material, making these mechanical gradients a powerful tool to control cellular phenotypes (**B**). Reprinted with permission from ref. [209]. Using melt-electrowriting combined with extrusion-based bioprinting of GelMA bioinks enables the layered distribution of various MSC mixtures (red, green and yellow) demonstrating a smooth transition between layers, which represents a key step towards more controllable and complex hierarchical structures (**C**). Reprinted with permission from ref. [212].

As the technological tools advance, the field is starting to move from biofabrication of stacked zones to high-resolution gradients, as illustrated in Figure 6A [192], to better mimic natural tissue hierarchies. The fabrication of gradients has been attempted with other fabrication techniques in past years, to replicate the anisotropic nature of biological tissues [193]. The involvement of rapid prototyping, and specifically biofabrication, herein holds huge potential to drive a more controlled production of gradients with higher resolution—and in high throughput. With detailed control over the implant architecture, powerful biological responses can be observed with imported mimicry of the extracellular anisotropic structure [194,195].

Designing scaffolds with pores is known to help facilitate tissue regeneration by increasing the diffusion of nutrients, waste and oxygen [27,196,197,198]. Depending on the size of the fabricated pores, porous implants can be used to for example improve cell growth due to a larger surface area for attachment [196,197], guide vascularisation through vessel invasion of larger pores [198], interloc with adjacent tissues as larger pores induce tissue ingrowth [199], or induceosteochondral formation before osteogenesis as small pores reduce local oxygen levels [199]. As native tissues are not homogeneously porous, the biofabrication of gradient pores has long been sought as it may guide multiple tissue types and interfaces to maximize overall performance. While most studies demonstrate improved cell seeding efficiency [200], uniformity [201], and mechanical properties [202,203] with gradient pore designs, Diloksumpan et al. reported decreased bone growth in 3D-printed gradient ceramic implants as compared to structures with constant porosity [204]. While exact pore size correlations may be difficult to make, it is clear that anisotropic architectures are able to modulate the regenerative process. However, to the best of our knowledge, there are currently no bioink examples with gradient pore sizes. This gap in the literature may be due to limited structural control offered by current bioinks. In addition to limited shape fidelity, the equilibrium swelling post printing and the changes in pore dimensions following possible enzymatic/hydrolytic degradation and cellular remodeling of the bioink matrix often yields dynamic changes in biofabricated pore sizes over time. Given the impact that pore size gradients have on biological responses, it is however important that the field also moves towards controlling and understanding this effect in bioinks in order to improve the clinical relevance of bioinks.

For bioinks, there is instead more of an interest to biofabricated structures with gradient mechanical properties to direct cellular differentiation, as this is easy to tune with current platforms. Although no biological components were included, Ober et al. were able to control the active mixing of two fluids to fabricate a continuous material at a microscale in 2015, setting the stage for bioprinting continuous gradients with a high resolution [205]. Freeman and Kelly were later able to leverage gradient stiffnesses in alginate-based bioinks to direct osteogenesis in the periphery of the constructs and adipogenesis in the softer core region of extruded fibres [54]. Similarly using extrusion based biofabrication, Nguyen et al. demonstrated that stiffer bioinks guided hypertrophic differentiation of stem cells while the soft surface of the tri-layered gradient constructs yielded less collagen type X and more collagen type II expression [206]. Exploring other biofabrication technologies to spatially vary mechanical microenvironments, Dobos et al. demonstrated that lithography-based 3D printing (two-photon polymerisation) could be used to print GelNOR in the range of 0.2–0.7 kPa [207]. They observed that cells encapsulated in softer regions started to migrate towards the stiffer areas of the inverse Gaussian-like structure [207], a phenomenon known as durotaxis [208]. Similar reports from Lavrentieva and colleagues also highlight increased migration for cells encapsulated in softer regions while further observing limited cell spreading and differentiation in stiffer GelMA bioink formulations, as seen in Figure 6B [209]. Interestingly, by using 3D-printed micromixers they were able to further generate a construct with combined cell and stiffness gradients [209]. While limited functional analysis was performed of the dual gradient structure, they were able to fabricate gradients with smooth transitions and overlapping interfaces which represents a sophisticated stepping stone towards more complex architectures with tidemark musculoskeletal regions.

The importance of moving from discrete gradients, with stepwise transitions, to smooth continuous profiles was demonstrated by Idaszek et al. [210]. Biofabricating a full-thickness structure (>5 mm) using alginate bioinks, uniaxial gradients of both cells and materials supported heterogeneous musculoskeletal tissue differentiation within one construct [210]. Although Chae et al. biofabricated a three layered tendon implant with discrete gradients using extrusion printing; after 9 weeks of implantation in rats, these implants were remodeled to constructs with smooth transitions between the bone–tendon interface [211]. By converging melt-electrowriting with the extrusion of bioinks, de Ruijter et al., were able to observe a smooth transition between three layers of different bioinks (mixing of components across zones) although it had been printed in a layer-by-layer process with 200–400 µm diameter bioink resolution (Figure 6C) [212]. It may be that this hybrid printing technology permits extrusion of low-viscosity bioinks that are able to fuse upon deposition. Interestingly, this exemplifies how continuous gradients may be achievable following post-fabrication/maturation steps, even in low-resolution scaffolds.

So, while there are a relatively limited number of studies that have successfully utilised biofabrication to generate high-resolution gradients, there is still a major interest in using this route to fabricate biomimetic hierarchical implants. However, there is a great need to develop robust characterisation techniques with high sensitivity in parallel, before structure to function relationships can be fully deciphered. Extensive work is needed to further move to submicron levels, providing reliable methods to mimic tissue heterogeneity at fibrillar levels.

## 4. Challenges in Clinical Translation

Although 3D bioprinting has paved the way to produce customised tissue-engineered structures [213], few technologies have yet been applied in widespread clinical settings. The ethical, safety and regulatory issues of translating 3D bioprinted tissue-engineered structures should be studied thoroughly. Biocompatibility, maintenance of aseptic conditions, and ex vivo manipulation present safety concerns, while ethical issues are correlated with ownership, cell harvesting, biomaterial, and commercialisation paths. From a regulatory perspective, the frameworks for bioprinted structures are not clear as they often span across several health areas (medical devices, biological drugs, regenerative medicine), and new robust guidelines are required to fit this purpose. Despite various studies on 3D-bioprinted tissues/organs, concerns associated with the translation characteristics have often been overlooked and a fit-for-purpose guidance is only starting to form [214]. “Technical Considerations for Additive Manufactured Devices” is one current guideline produced by the FDA in 2017 to show the design’s pathway, manufacturing considerations, and device testing matter for 3D-printed medical devices. While this provides a step in the right direction, recognising the unique testing requirements, this guideline is not good enough for biological products, and a complementary guidance is required for biofabricated constructs [215]. It has already been noted by the FDA that this framework will have to evolve as the understanding of both quality and safety of biofabrication grows. Others have also flagged that the single-patient-use customisation, which is one cornerstone of biofabrication to personalise medical treatments, may actually hold the ability to undergo exemptions from regulatory processes [216] and needs to be addressed further. In the sections below we discuss some of these challenges along with ethical concerns that need to be taken under consideration.

### 4.1. Regulatory Classifications and Governing Bodies

Biofabricated structures can be classified as biomedical products under a wider range of categories, such as drugs, biologics, devices, and even a combination category (including drug, biologics, and devices) [214]. In North America, the Food and Drug Administration (FDA; American organisation) guidelines should be followed for any human drugs, tissues, medical devices, and biological products. However, the Health Resources Services Administration is in charge of proving any developed organs, and vascularised organs are not regulated by the FDA [217]. One crucial question is thus to categorize tissue-engineered structures to find the proper regulation within a North American market. The FDA is conducting more research studies to find factors influencing the safety and quality, as there are no worldwide regulations for bioprinting [214,218]. In Europe, bioprinting is instead considered as part of the tissue engineering provisions and regulated as per its guideline [218,219]. The European Medical Devices Directive governs these complicated regulations. Other European organisations are the Active Implantable Medical Devices Directive, and the In-vitro Diagnostic Medical Devices Directive [219]. Depending on the type of device, 3D printing technology, software, and biomaterial, various regulations are implemented. Australia has changed their regulations as per the international definitions developed by the International Medical Device Regulations Forum [220], allowing low-risk devices to be mass-produced without any delay (regulating bioprinted tissues/organs as medical devices and out of the biologics category) [220]. All in all, there is no single guideline in terms of regulatory pathways and the need for a global pathway is essential.

### 4.2. Translational Pathways

The initial step in clinical translation is identifying the clinical needs to begin the design and development of a bioprinted tissue. This is followed by robust sterilisation, characterisation (chemical, mechanical, and physical properties), and in vitro/in vivo biocompatibility testing. Particularly, any animal study conducted in the US must be approved by the Institutional Animal Care and Use Committee at each local institution [214]. Good Manufacturing Practice and Good Laboratory Practice are both regulatory guidelines to preserve data integrity by reporting and monitoring, and quality by following predefined manufacturing standards [221]. In particular, the cell′s source, processing and manufacturing, biomaterial properties (source, quality, and biocompatibility), design control, and testing (mechanical and physical analysis) can be part of the preclinical studies to assess a tissue-engineered structure as per the regulations [217]. Additional steps include premarket evaluation and clinical trials. All these aspects are detailed in the sections below.

#### 4.2.1. Sterilisation

A factor which should be considered early is the sterilisation technique. An inevitable preclinical evaluation of any biofabrication technology study involved with cell incorporation or therapeutic applications is biomaterial sterilisation. Autoclaving (pressurised steam and high-temperature water) is used widely because it is relatively quick, efficient, and has a reasonable cost. This technique is not appropriate for biomaterials such as polysaccharides due to the effect of high temperature on them [222,223]. Gamma irradiation is another sterilisation method used chiefly for orthopaedic applications. It works based on transferring the energy from gamma radiation into the electrons of the biomaterial supposed to be sterilised [224]. UV works based on a similar principle to gamma irradiation, but it has lower energy photons [225]. It should be acknowledged that these standard sterilisation techniques can affect physiochemical biomaterial properties [225]. As a result, a biomaterial may not be printable after sterilisation. For instance, Hodder et al. showed that gamma irradiation could reduce the viscosity of alginate/methyl cellulose scaffolds due to the significant methyl cellulose molecular mass deduction affecting the chain mobility in calcium ions available at the alginate network. They reported ultraviolet (UV) and autoclaving as appropriate approaches, considering cell viability after sterilisation [225]. Other studies have shown that polymers degrade when exposed to UV light [226,227], which is likely the cause for the decrease of the elastic modulus in samples sterilised with UV irradiation. Similarly, bacterial degradation might alter the structural integrity of nonsterile hydrogel affecting the elastic modulus of alginate scaffolds [228]. It was also reported that other traditional methods such as lyophilization and ethylene oxide treatment affect the alginate structure negatively, as reported by Stoppel et al. [229]. Similarly, bacterial degradation might alter the structural integrity of nonsterile hydrogel affecting the elastic modulus of alginate scaffolds [228].

#### 4.2.2. In Vitro Evaluation

Biocompatibility is one of the crucial regulatory concerns to ensure that there is no adverse interaction between tissue and structure. The chemical composition, fabrication procedure, and sterilisation technique are evaluated. There are International Organisation for Standardisation (ISO) guidelines to evaluate the mentioned parameters appropriately [230]. Normally, as part of the biocompatibility examination, cytotoxicity, irritation, sensitisation, toxicity, and genotoxicity are assessed. As part of the assessment, the physical and mechanical properties are examined. For this, the American Society for Testing and Materials International (ASTMi), ISO, and the United States Pharmacopeia (USP) standards have protocols on how to evaluate physical properties [217]. Second, sterilisation plays a decisive role from a safety standpoint; the FDA, American National Standards Institute, Association for Advancement of Medical Instrumentation, and ISO have published regulations on sterilising medical devices using steam, ethylene oxide, dry heat, and ionising radiation [217]. However, not all sterilisation techniques are applicable due to the sensitivity of biological products, which is one of the challenges of using tissue-engineered constructs in human studies. Other techniques such as applying heat, acid, detergent, radiation, and chemicals are implemented instead [231,232]. Third, degradation is a significant concern; ISO-5840 offers a guideline to assess the degradation, but it is not comparable to a long-term human condition. Many studies approved the short-term mechanical stability of tissue-engineered structures, but long-term durability is still a challenge [233].

#### 4.2.3. In Vivo Evaluation

Animal studies are utilised extensively as preclinical studies to assess medical devices from a safety point of view. It is recommended to track and record covariables (e.g., cardiac rhythm, respiratory rate) on operative records if the animal study is acute (device-associated trends are expected to be transient). For chronic studies, the postimplant or postsurgical period should be carefully monitored as per laboratory research animals’ standards. At this step, all physiological information that is similar to the information obtained in human intensive care and recovery areas should be captured. Hence, any rationale behind an early failure or success can be explained. Additionally, to monitor pain and body temperature, stress variables can be controlled using established standard assessment paradigms. During interim observation periods, while animals are recovering from the surgical flow, animal weight information should be collected. Also, standard operating procedures should be used to collect clinical chemistry data. During the terminal study period, postmodern imaging methods, such as explant radiography (before histomorphometric analysis) and scanning electron microscopy (to characterise surface behaviours of implants), can be used. The histomorphometric analysis is another imaging technique used to properly interpret acute and chronic biologic responses and these may include mural injury, inflammation, vascularisation, intimal fibrin, and adventitial fibrosis. For further in vivo evaluation, local and downstream tissue assessment can be implemented to check for adverse observations, such as inflammation. As such, pathologic studies should include a systematic descriptive evaluation of the tissue [234].

Despite the valuable information acquired from such studies, these data cannot be reliable, as animal studies cannot precisely predict tissue-engineered structure functioning in humans. Hence, clinical outcomes in either clinical trials or premarket trials are not clear [235]. This is due to the difference in the anatomy of animals and humans, and second, the difference in the healing kinetics of humans and animals. Furthermore, other factors associated with human patients, such as exercise and genetic abnormalities, can influence the efficacy and postmarket evaluations. Various types of animals (e.g., dogs, pigs, calves, and sheep) have been utilised as standardised animal models [235]; however, none of these models replicate human anatomical and physiological conditions. This issue has yet to be investigated, and even ISO-5840 reports that there is no standard protocol to adhere to, due to various factors affecting animal studies (e.g., design and biomaterial). An animal care committee is in charge of considering ethical concerns for any studies involving animals. A similar committee called the Institutional Review Board does the same thing for issues involving human rights, privacy, and welfare. This institution is in charge of regulating, approving, and modifying clinical trials. The FDA is the agency that gives the translation pathway of tissue-engineered products in the United States. Other agencies worldwide also do so (e.g., European Medicines Agency in Europe, State Food and Drug Administration in China, and Therapeutic Goods Administration in Australia) [218].

#### 4.2.4. Premarket Evaluation

The next phase after obtaining preclinical approval is to begin premarket clinical investigations; in this step, safety and efficacy are studied before commercialisation. This is the time to examine whether the risk and potential advantages have been studied or not, by investigating the fabrication procedure, safety, and preclinical studies. Particularly, to translate a tissue-engineered structure and commercialise it, funding from industry, investors, or a capital firm is needed. Occasionally, the government supports research and development, such as the National Institutes of Health (NIH)

#### 4.2.5. Clinical Trials

Once moved into clinical trials, all products need to be evaluated further because of ethical concerns. These issues involve human rights and conflicts of interest. They mainly refer to the type of biomaterial or cell (clinical stage) and clinical trials and the consequences of the trials [236,237]. From a cell/tissue standpoint, the source of cells and donation is debatable [237,238]. Additionally, the ownership of human cells/tissues is not clear and is a subject of debate. The FDA has published a standard entitled “Human Cells, Tissues, and Cellular or Tissue-based Products” [239] to indicate how to create tissue-engineered structures by minimally influencing cells and their biological characterisation.

Concerning donors, more regulations are needed to protect donors′ privacy. Furthermore, once it is time to assess clinical trials, human safety is a most important ethical concern for in vitro and animal studies. There is no clear path for the clinical translation of tissue-engineered tissues/organs. In the United States, depending on the application, a tissue-engineered product can be examined by the FDA (e.g., implanted tissues), the Department of Health and Human Services (e.g., allograft), or even the surgeon (e.g., new surgical protocols). Good Clinical Practice processes should be in place for all clinical trials to confirm no violations of human subjects occur.

### 4.3. Other Considerations

With biofabrication offering an in-house use of technology, it should furthermore be considered how the fabrication of patient-specific implants or technologies at hospitals may raise additional safety concerns [240]. If this is instead outsourced in a strategic manner, both cost-effectiveness and safe transport and/or storability have to be investigated in detail [240]. The manufacturers have to further consider potential problems regarding the labelling and tracking of the constructs, which may cause difficulties with every implant being fully customised and unique. In addition to the implants themself, specialised surgical guides are often used in musculoskeletal medicine. The need for biocompatible 3D-printed materials as surgical guides is again less clear and requires an updated framework [240]. While personalised medicine is an attractive idea, it may be that the next era of biofabrication will focus more on stratified medicine patient cohorts over fully personalised medical devices to help overcome some of these regulatory hurdles by providing a more streamlined process.

## 5. Current Examples of Clinical Applications

While biofabrication is emerging as a new technology that is completely disruptive to the field of medicine, it is well recognised that several challenges persist before it can be translated to a clinical setting. There are however some great examples of how this technology can today, already make a difference to patients.

While the main interest in 3D models comes from cancer drug development and cosmetic toxicology testing, some models are also used today to screen drugs to treat osteoarthritis pain and slow the disease progression. Although still sparse, both cartilage-on-a-chip and joint-on-a-chip systems have emerged to offer increasingly complex biological systems for in vitro testing [241]. Using a bioreactor setup, Nichols et al. was able to combine several aspects of the joint microenvironment to study the administration of disease progressing cytokines as well as anti-inflammatory drugs [242]. Other studies have utilised soft lithography techniques to develop miniature cartilage devices that can also induce mechanical stimulation in a controlled manner, to study the effect on tissue regeneration [243,244]. Occhetta et al. further studied how anti-inflammatory drugs and commonly used bioink materials influence tissue repair [244]. While these exciting developments hold great potential to help advance the development of clinical drugs and new treatment options for osteoarthritis, they still lack full validation and thus implementation in today’s clinical setting. Beyond validating the efficacy and accuracy of these models, additional work will have to focus on the high-throughput use of these devices and how it can better resemble the whole joint space before it can see widespread use in drug development pipelines. However, the advantages of these models are that they are not meant to be implanted in a patient, and can thus follow other clinical translational pathways—which are still under development.

Biofabricated implants may furthermore be successfully applied to improve surgical training, planning and consultation. This type of application is not as heavily regulated as it has no direct patient use, it is simply a prototype strategy. Preoperative planning is already used widely for various surgeries to reduce surgery time and, most importantly, reduce the risk [245]. Recent studies show that 82 percent of preoperative planning had better outcomes using 3D-printed models than standard preoperative planning, with more than 50% of studies showing a significant decrease in surgery time [246].

In addition to proven intraoperative metrics, Jiang et al. highlight that several subjective benefits are recorded from both surgeons and patients due to increased intraoperative guidance and patient engagement, respectively [247]. As surgical simulations have revealed that more than 50% of errors are attributable to excessive force used during surgery, and it is not always possible to practice with real human bodies, using 3D-bioprinted tissue models may one day provide an excellent solution for preoperative planning [247]. For this purpose, many hydrogel biomaterials have been studied to provide better organ models for the preoperative surgery of soft tissues. Just like with any in vitro 3D-tissue model, there is however much left to learn, as the current technologies do not allow the full replication of mechanical properties critical to many musculoskeletal tissues [248], especially interfaces such as bone–tendon and muscle–tendon junctions [249]. With recent developments in fibre-oriented 3D structures [250,251], improved control over the modulus of elasticity [252,253], and high-resolution constructs moving towards anisotropic tissue quality [209,210,212,254,255], the field is progressively moving towards models that both feel and deform like real human tissue that can aid in both surgical training and planning.

## 6. Conclusions and Future Perspectives

This review paper firstly discusses the trend of currently available material strategies for musculoskeletal biofabrication and their advantages and disadvantages. This includes the mechanical, rheological, and cross-linking properties along with the cytocompatibility, cell viability, and printability and up-scaled sourcing of the biomaterials, identifying both clinical challenges and opportunities. With literature trends revealing a strong preference for naturally derived materials throughout the last decade, its exponential growth trend in publications denotes the progression of the biofabrication field beyond a structural integrity focus and towards more functional outputs. It is furthermore clear that many biomaterials have by now become well characterised and validated so that their advantageous properties can be harnessed across several platforms. This increased confidence that these materials can be fine-tuned to fit the specific purpose has further sparked the use of multimaterial bioinks to synergistically overcome limitations of the traditional biofabrication window.

Scientists have been able to reap the rewards from these base material developments by showcasing several sophisticated strategies to print low-viscosity bioinks, overcoming the delicate balance of printability and without having to compromise functionality. Recent work highlights the move to complex multimaterial bioinks that also contain growth factors and other nutrients crucial to guiding the musculoskeletal tissue regeneration process. By combining clever biomaterial platforms with cutting-edge bioprinting technologies, several studies are now demonstrating good therapeutic effects without having supraphysiological doses, due to sophisticated spatial- and temporal-delivery control. This updated delivery control further extends to cells, which enables the building of hierarchical structures with smoother, high-resolution, zones. While these gradient structures have proven the potential for users to direct both cellular migration as well as differentiation, understanding what this new high-resolution control is fully capable of is still a bit of an enigma, as few studies have successfully achieved this to date. So, as these technological advances are being made, we invite researchers to include more functional outputs to drive this new era of biofabrication. Additional work is further needed when it comes to the awareness of both the ethical and translational pathways, specifically regarding the evolution of technological advances made and the current lack of a clear path for biofabricated tissues and organs.

So, while the field of biofabrication holds the potential to fully disrupt health research as we know it, the field will have to focus on specific aspects in order to reach efficacy in a clinical setting. This includes, but is not limited to (1) understanding batch-to-batch variability and robustness of multimaterial bioink formulations that contain more than two-components, (2) balancing the paradoxical need for soft hydrogels to regenerate new tissue while maintaining compressive strength similar to the native range of musculoskeletal tissues—several magnitudes higher, (3) managing endotoxin levels and antibody reactions of bioinks and biomaterial inks, (4) improving the evaluation methods of successful bioink- and tissue-anisotropy, (5) recapitulating soft–hard tissue interfaces that occur within native musculoskeletal organs, (6) understanding structure-to-function relationships that promote host-tissue integration, and (7) navigating and updating the regulatory pathway—assessing how novelty can yield undefined benefits to patients while providing a unique risk profile.

As the field of biofabrication works towards this improved functionality, delivery and resolution control, the utilisation of the technology to fabricate 3D models and preoperative planning tools offers a unique opportunity to learn more about how 3D-printing processes, and subsequently biofabrication, works in a clinical setting. Although many commercial challenges still remain, the field of biofabrication can mirror many of the logistical aspects of setting up cell-free 3D-printing facilities within hospitals. By looking at the implementation of 3D-printing within hospitals, the field of biofabrication can evaluate the efficiency of strategies outsourcing the technologies off-site, and further study the ethical aspects of storing patient information and tissue samples as well as the practical aspects of working together with clinicians and patients to ultimately revolutionise the health care system.

## Data Availability

Publicly available datasets were analyzed in this study. This data can be found here: www.scopus.com.
